# Lung adenocarcinoma with eosinophilic pleural effusion: A case report

**DOI:** 10.1097/MD.0000000000027982

**Published:** 2021-12-03

**Authors:** Xiangxiang Zhou, Jingxuan Wan, Xin Gan

**Affiliations:** a Department of Respiratory and Critical Care Medicine, Jiangxi Provincial Chest Hospital, Nanchang, Jiangxi, China; b Department of Pulmonary and Critical Care Medicine, Center of Respiratory Medicine, China-Japan Friendship Hospital, Beijing, China.; c The First Affiliated Hospital of Nanchang University, Nanchang, China.

**Keywords:** eosinophilic pleural effusion, lung adenocarcinoma, malignancy, tuberculosis

## Abstract

**Rationale:**

Eosinophilic pleural effusion (EPE) is a rare phenomenon in which the etiological diagnosis remains a challenging issue; here, we present a patient who was eventually diagnosed with malignant EPE by parietal pleural biopsy.

**Patient concerns:**

The patient was a 73-year-old man with pulmonary tuberculosis who was taking isoniazid and rifampin; after 6 months, he had right-sided eosinophilic pleura, and histopathological examination of the parietal pleura revealed malignant cells from the lung.

**Diagnosis:**

Based on the parietal pleural biopsy, the patient was diagnosed with lung adenocarcinoma with ipsilateral pleural metastasis stage IVA.

**Interventions:**

The patient received a first-line systemic chemotherapy regimen (premetrexed and carboplatin).

**Outcomes:**

The patient received 2 cycles of chemotherapy, and based on the response evaluation criteria for solid tumors, he achieved partial response and the effusion disappeared.

**Lessons:**

This case presents a patient with tuberculosis who was suffering from an EPE, which was eventually diagnosed as malignant EPE based on histopathological examination through medical thoracoscopy, although multiple Thinprep cytology tests showed no evidence of malignancy, pleural biopsy is necessary to obtain an accurate etiology diagnosis.

## 1 Introduction

Eosinophilic pleural effusion (EPE) is defined as pleural inflammation that contains at least a 10% eosinophil count in the pleural effusion.^[1]^ These effusions occur rarely and the etiologies are not well known, and a meta-analysis of 687 cases in 2012 showed that EPE was most often due to malignancy, followed by idiopathic, tuberculosis (7%), and other causes.^[2]^ In addition, different causes have been reported owing to the increasing number of case reports and series researches.^[3,4]^ Here, we present a case of malignant eosinophilic pleurisy that was distinguished from tuberculosis EPE by pathological examination after three-time pleural cytology and parietal pleural biopsy through medical thoracoscopy; we hope that this case can provide a better understanding of the different causes of EPE and facilitate rapid diagnosis leading to accurate treatment.

## 2 Case presentation

A 73-year-old man was admitted to our hospital with progressive cough for 1 month, respectively, in February 2020. The patient had a dry cough and was absent from fever, chest pain, or weight loss. He was diagnosed with pulmonary tuberculosis 6 months ago and was taking oral isoniazid and rifampin regularly. The patient was a farmer without occupational exposure to asbestos and inhaled irritants. He had a 52 pack-year smoking history and denied recent chest trauma, surgery, or other medicine problem. The physical examination revealed dullness and decreased breath sounds throughout the lower half of the posterior right side of the chest and no other significant abnormality.

**Figure 1:**
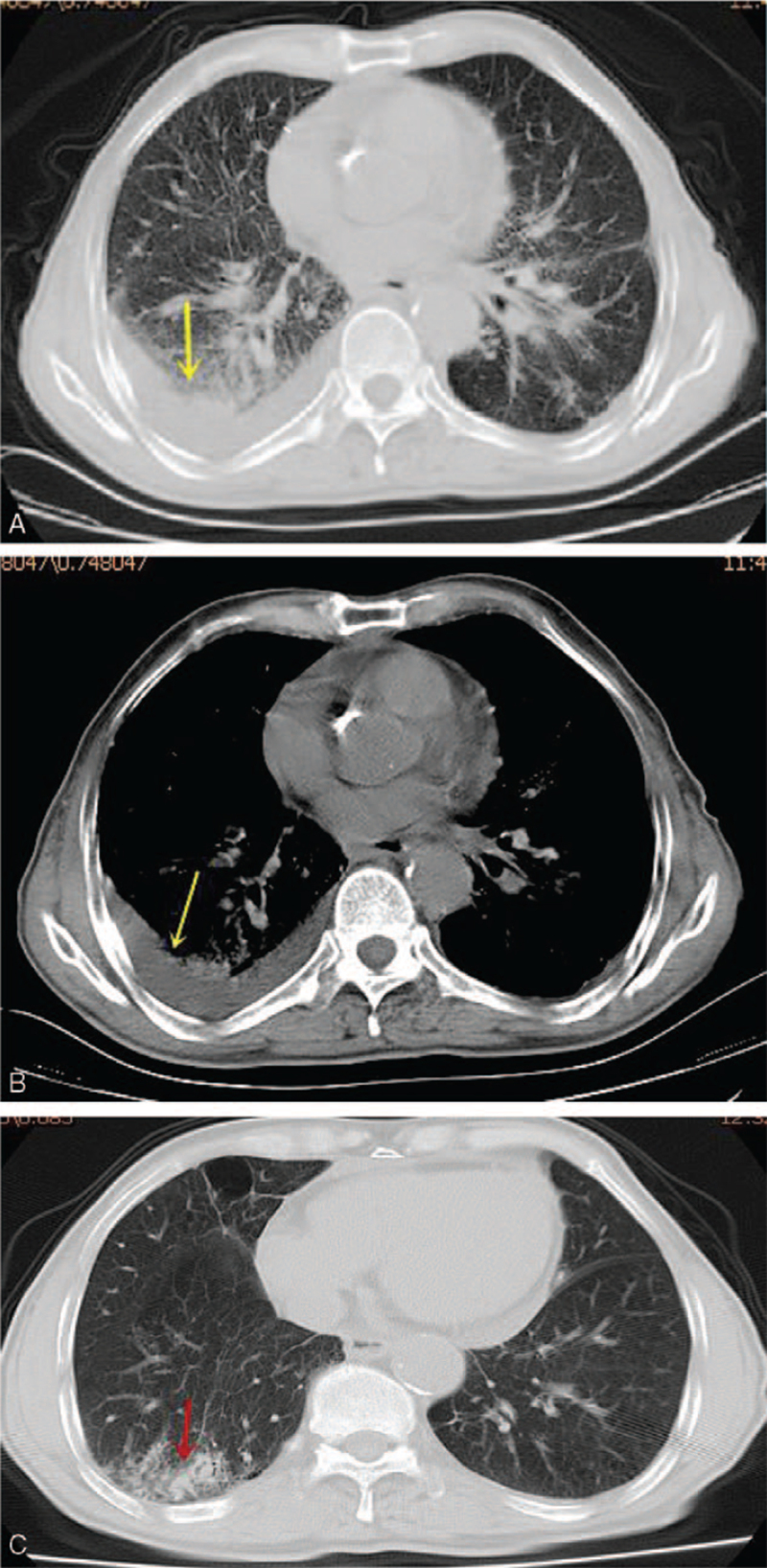
CT scan results. (A, B) The right-sided pleural effusion with adjacent pulmonary atelectasis (yellow arrow). (C) The effusion disappeared (red arrow) since the patient completed 2 cycles of chemotherapy. CT = computed tomography.

Routine laboratory examination indicated a peripheral blood leukocyte count of 5700/μL (neutrophils 78.3%, lymphocytes 11.10%, and 2.1% eosinophils) and an elevated erythrocyte sedimentation rate (107 mm/h). The carcinoembryonic antigen 12-5 and carcinoembryonic antigen 15-3 were mildly high, and computed tomography revealed right-sided pleural effusion with adjacent pulmonary atelectasis (Fig. 1A and B). The thoracentesis revealed the straw-colored fluid with exudative pattern of eosinophils (80%), lymphocytes (20%) in the leukocyte differential count. Total protein value of the pleural fluid was 41.1 grams per liter and the elevated carcinoembryonic antigen 12-5 was 597.40 U/mL. The adenosine deaminase (ADA) level of the pleural fluid was 9.0 U/L and tuberculosis antibody was positive. The pleural fluid culture was negative for tuberculosis or mycobacterium. Cytological examinations of pleural fluid conducted three times showed no malignant cell but significant eosinophilic infiltration with some lymphocyte and mesothelial cells observed. The serum of autoantibodies (including antinuclear antibody) and complements were negative, which did not suggest collagen vascular disease. The parasitic disease was ruled out because the patient showed normal serum cytomegalovirus antibodies, toxoplasma antibody, and immunoglobulin E level.

**Figure 2:**
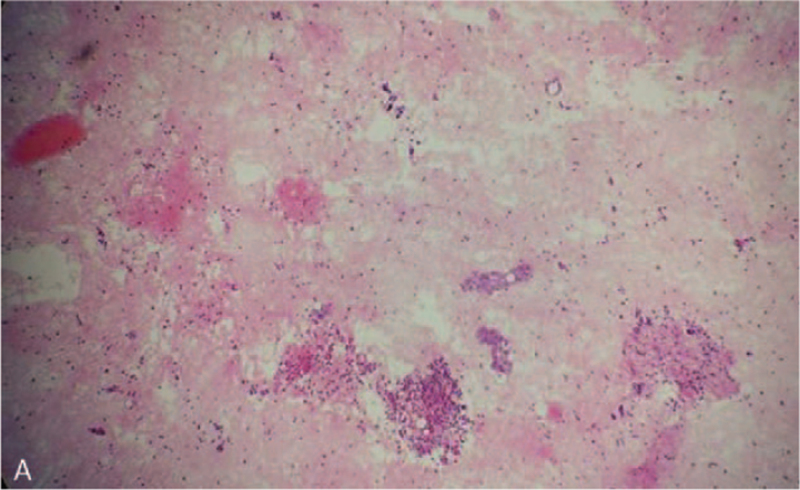
The pathological examination of the right parietal pleural biopsy. (A) The atypical cells formed cord-like and nest-like pattern.

Tuberculosis has been declared to be the common cause of EPE; the initial diagnosis upon admission in our case was pulmonary tuberculosis with EPE, the Herxheimer reaction during the course of chemotherapy for tuberculosis and drug-resistant pulmonary tuberculosis could be excluded; the patient maintained oral isoniazid and rifampin, and oral prednisone (0.5 mg/kg/d) with the dose tapered sequentially; however, the pleural effusion persisted and there was no relief of the dry cough after two weeks. Medical thoracoscopy was performed with positive cooperation, and pathological examination of the right parietal pleural biopsy revealed that atypical cells formed cord-like and nest-like patterns under the microscope (Fig. 2A). Immunohistochemical analysis showed positive staining for NapsinA, thyroid transcription factor-1, cytokeratin 7 and negative for cytokeratin 20, cytokeratin 5/6, P63, carcino-embryonic antigen, Syn, and Wilm tumor gene 1. The Ki-67 expression was 50%. Histopathological examination and immunohistochemistry revealed that the atypical cells were glandular epithelial cells from the lung, and the patient was clinically diagnosed with lung adenocarcinoma with ipsilateral pleural metastasis stage IVA, and informed written consent was obtained from the patient for the publication of the case report.

According to the first-line chemotherapy for advanced non-small cell lung cancer, the patient received pemetrexed (500 mg/m^2^ on day 1) and carboplatin (area under the curve = 5) on day 1 every 3 weeks for 4 cycles. The patient completed 2 cycles of therapy, the effusion disappeared (Fig. 1C). According to the response evaluation criteria in solid tumors, the patient achieved partial response. There was no emerging effusion or metastasis after a follow-up of 6 months, and adverse events were evaluated according to the Common Terminology Criteria for Adverse Events, and the patient displayed symptoms including nausea and vomiting, and the toxicity grade was grade 2.

## 3 Discussion

EPE is rarely encountered in clinical practice and accounts for 10% of all pleural effusions.^[2]^ EPE was initially described by Harmsen in 1894.^[5]^ Since then, most information about EPE comes from small series and case reports. The cause of EPE is a diagnostic challenge for the clinical physician. There were 14% to 25% of EPEs remain undiagnosed and they are called “idiopathic.”^[6]^

Chest traumas including pneumothorax, haemothorax, thoracotomy, repeated thoracentesis, or thoracoscopic surgery are frequently associated with EPE. The presence of air or blood in the pleural space acts as a stimulant for eosinophilic pleuritis. However, our patient denied pleural trauma or surgical procedures. In addition, EPEs are likely to be associated with connective tissue disease, infection resulting from bacteria, mycobacteria, parasites, fungi, and pulmonary thromboembolism,^[4,7]^ and in our case, the autoimmune profile, the relevant clinical and laboratory findings were normal. Recently, certain drugs containing warfarin, pirfenidone as well as methimazole are recently reported that are related to the development of EPE,^[8–10]^ but our patient was taking oral isoniazid and rifampin for 6 months and denied other suspicious drugs intake.

The relationship between tuberculosis and EPE was constantly updated, and it was reported that tuberculosis pleural fluid scarcely contains high eosinophils, and one study stated that one could exclude tuberculosis if eosinophils were found in the pleural fluid with a significant number in 1997.^[11]^ Our patient was diagnosed with tuberculosis and had already accepted therapy for 6 months. The tuberculous EPEs are expected to have a high ADA levels. Conversely, the pleural fluid ADA level of our patient was within the normal range. Malignancy was preliminarily excluded since the three-time cytological examinations showed no tumor cell in the pleural fluid. Benign idiopathic EPE is a diagnose of exclusion and respond dramatically to corticosteroids,^[6]^ but our patient showed a poor response.

The probability of malignancy is inversely related to the percentage of eosinophils in the pleural effusion,^[1]^ which is inconsistent with our case; although there were 80% eosinophils in the pleural effusion, even the 3-time pleural cytologies showed no malignant cells, the pleural biopsy turned out to be adenocarcinoma; therefore, malignancy, such as lung cancer and mesothelioma, is a critical etiology that should be considered in patients with EPE.^[12]^ In addition, pleural biopsy through medical thoracoscopy or open thoracoscopy is urgently recommended, especially for patients with eosinophil-rich fluid persisting or the cause remaining unclear. Previously study showed that host-tumor cell interactions contribute to EPE,^[13]^ and eosinophils play a pleiotropic role in the tumor microevironment.^[14]^ Some studies have suggested that cancer patients can benefit from tumor-associated eosinophilia, resulting in a modest survival.^[15]^ The functional role of eosinophils in malignant tumors remains to be clarified in the future.

## 4 Conclusion

There are different causes of EPE and making an accurate diagnosis in an individual case still remains a challenging issue, and our case emphasizes that pleural biopsy is indispensable to make a correct diagnosis, especially in diagnostically challenging cases, and careful follow-up is needed for an idiopathic EPE.

## Author contributions

**Conceptualization:** Xiangxiang Zhou.

**Investigation:** Xiangxiang Zhou.

**Project administration:** Xin Gan.

**Supervision:** Jingxuan Wan, Xin Gan.

**Writing – original draft:** Xiangxiang Zhou, Jingxuan Wan.

**Writing – review & editing:** Xiangxiang Zhou, Jingxuan Wan.
